# Angiogenesis Inhibitors and Immunomodulation in Renal Cell Cancers: The Past, Present, and Future

**DOI:** 10.3390/cancers14061406

**Published:** 2022-03-09

**Authors:** Lawrence Kasherman, Derrick Ho Wai Siu, Rachel Woodford, Carole A. Harris

**Affiliations:** 1 Department of Medical Oncology, St. George Hospital, Kogarah, NSW 2217, Australia; derrick.siu@sydney.edu.au (D.H.W.S.); rachel.woodford@health.nsw.gov.au (R.W.); carole.harris@unsw.edu.au (C.A.H.); 2 St. George and Sutherland Clinical Schools, University of New South Wales, Sydney, NSW 2217, Australia; 3 Department of Medical Oncology, Illawarra Cancer Care Centre, Wollongong, NSW 2500, Australia; 4 National Health Medical Research Council Clinical Trials Centre, University of Sydney, Camperdown, NSW 2050, Australia; 5 Faculty of Medciine and Health, University of Sydney, Camperdown, NSW 2050, Australia

**Keywords:** renal cell carcinoma, immunotherapy, angiogenesis inhibitor, targeted therapy, drug resistance, tumor microenvironment

## Abstract

**Simple Summary:**

In their advanced stages, the mainstay of kidney cancer treatment is with medications such as targeted or immune therapies. Breakthroughs in scientific understanding of cancer drug development have led to substantial improvements in life expectancy. Although several combinations are available to choose from, it remains unclear which is best, and furthermore why cancers become resistant to treatment. This review article explores the scientific basis behind drug treatments in kidney cancers, with particular focus on blood vessel development and the immune system, and summarizes the available evidence supporting multi-drug treatments in this context.

**Abstract:**

Angiogenesis inhibitors have been adopted into the standard armamentarium of therapies for advanced-stage renal cell carcinomas (RCC), but more recently, combination regimens with immune checkpoint inhibitors have demonstrated better outcomes. Despite this, the majority of affected patients still eventually experience progressive disease due to therapeutic resistance mechanisms, and there remains a need to develop novel therapeutic strategies. This article will review the synergistic mechanisms behind angiogenesis and immunomodulation in the tumor microenvironment and discuss the pre-clinical and clinical evidence for both clear-cell and non-clear-cell RCC, exploring opportunities for future growth in this exciting area of drug development.

## 1. Introduction

**Figure 1:**
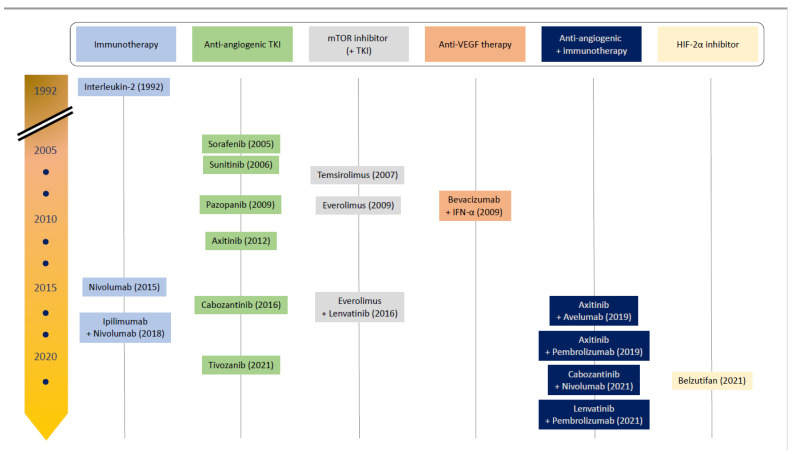
Timeline of US Food and Drug Administration drug approvals for advanced-stage renal cell carcinoma. Initial clinical success was seen with targeted therapies affecting angiogenesis and PI3K pathways in 2005, until 2015 when the first immune checkpoint inhibitor was approved for use. Within the last five years, more successes have been experienced with combination therapies, suggesting therapeutic synergy. Belzutifan is only approved for von Hippel–Lindau-related clear-cell renal cell carcinomas. Abbreviations: IFN-α = interferon alpha; TKI = tyrosine kinase inhibitor; mTOR = mammalian target of rapamycin; VEGF = vascular endothelial growth factor; HIF-2α = hypoxia-inducible factor-2α.

In their advanced stages, renal cell carcinomas (RCC) remain highly lethal, with over 13,000 deaths in the United States annually [1]. Historically, cytotoxic agents have been ineffective in treating RCC. Prior to 2007, when sunitinib was introduced, aldesleukin (interleukin-2) was the only US Food and Drug Administration (FDA)-approved agent for metastatic renal cell carcinoma. However, within the last 15 years, the treatment of renal cell carcinomas has completely changed with the introduction of a plethora of anti-angiogenic tyrosine kinase inhibitors (TKIs) and immune checkpoint inhibitors (ICI) into standard practice (see Figure 1), driven by a surge in understanding about renal cell carcinogenesis in the scientific community. Recently, larger-scale clinical trials in clear-cell RCC (ccRCC) have demonstrated favorable outcomes in combining these two drug classes [2,3,4], and several other trials are currently underway.

Table Despite recent advances, it remains clear that a substantial proportion of patients will either not respond or become resistant to treatment after initially responding, signifying a need to improve on current knowledge of drug resistance and therapeutic synergies. Furthermore, due to the rarity of diagnoses, non-clear-cell RCC (nccRCC) subtypes remain grossly understudied, resulting in a paucity of evidence to guide optimal management. Further study delving into the role of angiogenesis and immunomodulation may be the key to improving outcomes further for patients with advanced RCC. In this review, we discuss the underlying pathogenetic processes involved in RCC carcinogenesis, focusing particularly upon angiogenesis and immune dysregulation as potential therapeutic targets; review existing scientific and clinical evidence for combining these drug classes; and extrapolate upon potential innovations that are underway in this area.

### 1.1. Angiogenesis Is a Hallmark of Cancer Development

Throughout the course of transformation from a normal cell to a cancer cell, several malignant processes must occur, termed the “Hallmarks of Malignancy” in a seminal essay in 2000 [5]. At this time, the authors identified six features of tumorigenesis: insensitivity to growth-inhibitory signals, limitless replicative potential, self-sufficiency in growth signals, evasion of apoptosis, sustained angiogenesis, and tissue invasion and metastasis [5,6]. These were later refined to include immune escape and reprogramming of energy metabolism, as well as enabling characteristics of genomic instability and tumor-promoting inflammation [7], in light of further research and an increasing understanding of the role of the tumor microenvironment [7,8]. Each of these processes is multifaceted and complex, with significant clonal heterogeneity [9].

All cells must reside within 100–200 µm of a capillary vessel, and pro-angiogenic signals are required to form new vessels as tumors increase in size [5,9]. In the absence of access to adequate vasculature and, consequently, lack of nutrients and oxygen [7,10], tumor cells become necrotic or apoptotic [11,12,13], and tumor growth cannot progress beyond a critical size [9]. In existing literature, “angiogenesis” refers interchangeably to all forms of neovascularization or the process of vascular sprouting, cell division, migration, and assembly of endothelial cells (EC) from pre-existing vessels [7] Throughout this article, angiogenesis will refer to the latter.

The term “angiogenic switch” is used to refer to the balance of pro- and anti-angiogenic signals that control angiogenesis [5]. This balance involves pro-angiogenic signals through *VEGF-A* gene activation, the fibroblast growth factor (FGF) family of receptors, and matrix metalloproteases and is counteracted by anti-angiogenic signals such as thrombospondin-1. Following embryonic vasculogenesis and angiogenesis, dual processes through which endothelial cells differentiate from mesenchymal precursors and then subsequently expand through sprouting or intussusception [14,15,16], these processes are activated only transiently in normal tissues through the balance tipping in favor of pro-angiogenic factors for female reproduction and wound healing [9,17]. In tumors, this balance is constitutively active through mechanisms of increased production of pro-angiogenic factors and down-regulation of inhibitors to produce new blood vessels or co-opt existing ones [5,7,11]. This situation includes a complex interplay of genetic and environmental factors in which the tumor microenvironment plays a significant role and is yet to be completely understood [9,14]. One important environmental factor stimulating VEGF and VEGFR expression is hypoxia, signaling through the hypoxia-inducible factor-1α (HIF-1α) [18]. This is a pathway of increasing importance in renal cell carcinoma, and it will be discussed in subsequent sections [19].

Tumoral blood vessels are typically aberrant, displaying abnormal structure, function, and organization [20]. These vessels branch irregularly, follow a tortuous course, are associated with arteriovenous shunting, and have an altered surface area to volume ratio [20]. These properties often result in chaotic blood flow and areas with higher concentrations of carbon dioxide, resulting in an acidic microenvironment [9,21]. Tumor vessels are often hyperpermeable, or “leaky”, a feature that leads to extra-vascular clotting and fibrin deposition as well as abnormal angiopoietin-2 (Ang-2) expression or Ang-1 suppression, contributing further to angiogenesis [22,23].

In addition, tumor vasculature lacks normal protective mechanisms such as functional perivascular cells, required for the protection of vessels against changes in hormonal balance or oxygen levels, and tumor vessel walls may often include cancer or endothelial cells [9,24]. These vessel walls non-uniformly express adhesion molecules, impairing the ability of activated lymphocytes to adhere to tumor vessels, with implications for anti-tumor immune activity [9,25].

Vascular mimicry has also been described in tumors, referring to tumor cells forming vessel-like structures. This is a typical feature of aggressive malignancies such as melanoma and contributes to tumor invasion, cell motility, and metastasis [18,26,27]. Consequently, tumoral vessels do not respond to alterations in blood flow in conventional ways [28].

The degree of tumor neovascularization has prognostic implications, as tumor aggressiveness has been correlated with high expression of pro-angiogenic factors [29]. Furthermore, all the above features affect delivery of therapeutics to tumor tissue as well as efficacy, rationalizing the therapeutic combination of angiogenesis suppressants or receptor inhibitors with cytotoxic chemotherapy or immunotherapies [29].

### 1.2. Immunomodulation and Angiogenesis: A Crucial Interface in Renal Cell Carcinomas

The immune system interacts with the tumor at multiple stages of development. The theory of immune surveillance is that the tumor interacts with the host immune system in three distinct stages, elimination, equilibrium, and escape [30]. In the elimination stage, nascent tumor cells are eradicated by the immune system. Equilibrium refers to immune control over tumor expansion and metastasis until certain tumor cells develop mechanisms to resist the immune system and immune escape occurs [30]. Refining upon this is the concept of immunoediting, wherein the immune system selects for tumor cells of reduced immunogenicity or antigenicity, facilitating the eventual tumor immune escape [30,31]. Contributing to immune escape are tumoral recruitment of immunosuppressive leukocytes [31] and generation of a resistant microenvironment that suppresses productive anti-tumor immunity [32].

Increasingly, it has been recognized that tumors are more than a collection of heterogenous neoplastic cells; they contain infiltrates of immune cells as well as ostensibly normal cells recruited to provide structure and nutrition to the growing tumor [7,33,34]. Furthermore, interactions between the tumor cells and surrounding components serve to further tumorigenesis, metastasis, clonal evolution, epithelial–mesenchymal transition, and invasion [35,36]. This surrounding milieu is referred to as the tumor microenvironment and is of paramount importance in therapeutic development as recognition of the ability of the tumor microenvironment to shape responses to therapy and drug resistance grows.

Cells of the tumor microenvironment include immune inflammatory cells, pericytes, endothelial cells, adipocytes, lymphatic vessels, and cancer-associated fibroblasts, the latter of which serve to produce extracellular matrices with resultant tumor desmoplasia [36,37]. While this surrounding stroma was long thought to be the reaction to the presence of cancer with recognized therapeutic implications [38], more recently, it has been recognized as a complex interaction between the tumor cell and the host [36]. The immune cells that reside in the tumor microenvironment include lymphocytes, macrophages, and polymorphonucleocytes [39], many of which migrate into the tumor through the generation of tumoral chemo-attractants such as CSF-1, IL-3, and VEGF and chemokines such as CCL-2 [40,41,42].

Tumor-infiltrating inflammatory cells have both tumor-promoting and tumor-antagonizing effects. Monocytes have a role in tumor initiation through pro-inflammatory action on tissues; however, in the established tumor, they differentiate into an inactivated “trophic” phenotype, promoting tissue growth, production of growth factors and proteases facilitating metastasis, and suppression of T-cell signaling and responses [40,43]. Additionally, tumor-associated macrophages accumulate in areas of hypoxia and can contribute to pro-angiogenesis in these areas [5,7,11,44]. The process through which this change in macrophage phenotype occurs is poorly understood [40,41]. These suppressive macrophages, often termed “myeloid-derived suppressor cells”, may migrate to local lymph nodes, presenting tumor cells in a manner suppressive to T-lymphocytes through the use of programmed cell death-ligand-1 (PD-L1), PD-L2, IDO, and regulatory T cells (Treg) [43]. Other myeloid cells observed in the tumor microenvironment include tumor-associated neutrophils and tolerogenic dendritic cells, which contribute to immunosuppression and chronic inflammation through production of inflammatory mediators, further perpetuating a microenvironment hostile to activated immune cells and antigen-presenting cell maturation [42,44].

Tregs are immunosuppressive cells mainly derived from CD4^+^ T cells and defined by FoxP3 expression [42]. These cells function in the healthy host to maintain self-tolerance and immune homeostasis through a variety of mechanisms including immune checkpoints, metabolic disruption of T effector cell (Teff) activity, release of suppressive cytokines, promotion of T cell exhaustion and expansion, and direct cytotoxicity [42]. In tumors, they promote immunosuppression within the tumor microenvironment through upregulation of immune checkpoint molecules, enhancement of Treg generation, and promotion of tolerogenic dendritic cells with an impaired ability to activate Teff and cytotoxic T lymphocytes [42,45]. Tolerogenic DCs in turn also promote Treg function and generation in the tumor microenvironment [45].

As an extension of the complex interaction between the host immune system and the tumor, the composition of the tumor microenvironment has prognostic implications on clinical outcomes. Intratumoral lymphocytes (TILs), comprising CD4^+^, CD8^+^, and occasionally B and natural killer (NK) cells [46], are a major prognostic factor in numerous solid tumors, suggesting improved outcomes compared to those with low lymphocytic infiltrates [30,47,48,49]. In contrast, higher intratumoral Treg to T effector cell ratios are associated with poorer prognoses [50]. The composition of the microenvironment is thought to explain differences in response to single-agent immunotherapy agents, and an increasing recognition of the plasticity of the tumor microenvironment and emergence of immune escape variants provide an argument for combination therapy use [51].

**Figure 2:**
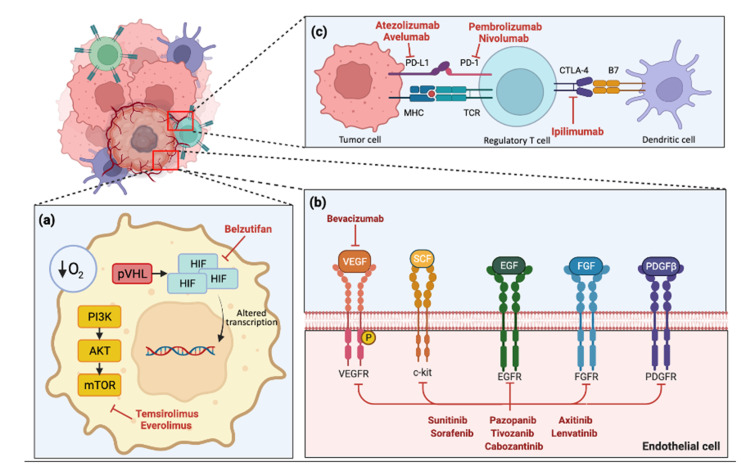
Schematic diagram of tumor environment in renal cell carcinoma with currently available therapeutic drug targets. Created with Biorender.com. (**a**) Intratumoral cell hypoxia leads to PI3K pathway pVHL activation, leading to HIF accumulation and altered cellular transcription, with downstream effects including angiogenesis factor production. (**b**) Various receptors and pro-angiogenic factors that interact with endothelial cells. (**c**) Immune checkpoints on regulatory T cells such as PD-1 and CTLA-4 are integral to the anti-tumor response, and their inhibition allows T cells to activate and cause cell death.

Renal cell carcinoma has long been recognized to be profoundly immunogenic, displaying high levels of T-cell infiltration and immune infiltrates [52,53]. However, the latter includes defined groups of immunosuppressive tumor-associated macrophages, providing a rationale for the relatively small response rate seen with single agent immunotherapy [53,54,55]. As previously alluded to, VEGF has a suppressive effect on immunity, and the process of neo-angiogenesis—in part governed by VEGF expression—allows for the accumulation of myeloid-derived suppressor cells and Tregs [56]. Inhibition of angiogenesis therefore can have notable effects on the immunosuppressive tumor microenvironment, making the combination a powerful anti-neoplastic strategy in advanced renal cell carcinoma (Figure 2).

Abbreviations: PD-L1 = programmed cell death ligand-1; PD-1 = programmed cell death-1; MHC = major histocompatibility complex; TCR = T-cell receptor; CTLA-4 = cytotoxic T-lymphocyte-associate protein-4; pVHL = Von Hippel-Lindau protein; HIF = hypoxia inducble factor; PI3K = phosphoinositide 3 kinase; AKT = serine/threonine kinase AKT; mTOR = mammalian target of rapamycin; VEGF = vascular endothelial growth factor; SCF = stem cell factor; EGF = endothelial growth factor; FGF = fibroblast growth factor; PDGF = platelet derived growth factor; VEGFR = vascular endothelial growth factor receptor; c-kit = proto-oncogene c-kit; EGFR = endothelial growth factor receptor; FGFR = fibroblast growth factor receptor; PDGFR = platelet derived growth factor receptor.

### 1.3. Drug Development of Advanced Clear-Cell Renal Cell Carcinoma

Systemic therapy is the mainstay of treatment in advanced ccRCC, and the treatment landscape has evolved significantly in recent decades (Figure 1). The introduction of VEGF inhibition led to significantly improved outcomes [57,58,59] and, more recently, ICI [55], especially when used in combination with either other ICI [60] or VEGF TKIs [2,3,4], have continued to show improved outcomes for patients. This section reviews the history of drug development in ccRCC, leading up to the success of combinations that capitalize on the therapeutic synergy between tumor angiogenesis mechanisms and the immune system.

#### 1.3.1. Cytokine Therapy

Traditionally, RCCs were thought to be chemoresistant, with documented overall response rates (ORR) < 10% [61,62]. In view of observed spontaneous tumor regression and response to cytokine therapies in one cohort study [63], immune mechanisms were suggested to play a significant role in the pathophysiology of RCCs [64]. To address this, pre-clinical work focused on the potential activity of IL-2 and IFN-α.

IL-2 was thought to activate NK cells to secrete cytokines which potentiate monocyte and macrophage activity while also increasing NK-cell lytic capacity, accounting for the lymphokine-activated killer cell phenomenon leading to its use as an immune stimulant [65]. Similarly, IFN-α has immunomodulatory effects on dendritic cells which induce T- and B-cell immunity and also demonstrates anti-tumor properties via induction of apoptosis and inhibition of cell growth [66]. In the 1990s, these therapies were studied and demonstrated very modest survival benefits in patients with advanced RCC; however, these were associated with significant toxicities [67,68].

#### 1.3.2. VEGF and Other Angiogenesis Inhibitor Monotherapies

Subsequent pre-clinical work identified that, in the majority of sporadic ccRCC cases, alterations of the von Hippel-Lindau (VHL) gene by deletion, mutation, or methylation led to inactivation, causing upregulation of HIF-α and increased production of VEGF and platelet-derived growth factor (PDGF), promoting tumor angiogenesis, growth, and metastasis [69,70,71]. This understanding led to the use of anti-angiogenic strategies in the treatment of ccRCC (Figure 2b).

In 2003, bevacizumab, a monoclonal antibody against VEGF, was demonstrated in a prospective phase II study to have clinical activity in ccRCC [59]. Following this, several anti-angiogenic oral tyrosine kinase inhibitors (TKIs) emerged in the treatment landscape of advanced ccRCC. Sorafenib, an oral multitargeted TKI against VEGFR-1, -2 and -3, platelet-derived growth factor receptor β (PDGFR-β), FMS-like tyrosine kinase (Flt-3), c-Kit protein, and RET-receptor kinases, was tested in a phase III placebo-controlled study in patients as subsequent-line therapy [72,73]. Sorafenib was associated with benefit in progression-free survival (PFS) (5.5 vs. 2.8 months), but no overall survival (OS) benefit was demonstrated likely due to substantial crossover. In another randomized phase II study, sorafenib was compared to IFN-α-2a as first-line treatment in ccRCC; however, no demonstrable benefit in PFS was seen [69]. In the pretreated ccRCC setting, other multi-targeted TKIs have also demonstrated efficacy in large randomized studies including tivozanib, axitinib, and cabozantinib [70,71,74].

Subsequently, two multi-targeted TKIs were assessed in the first-line advanced-stage ccRCC setting in large, randomized trials meeting their primary survival endpoints, and were approved by the FDA [57,58]. Sunitinib, an oral TKI against VEGFR and PDGFR, demonstrated significant benefits in a phase III study of 750 patients over IFN-α-2a in terms of PFS (HR 0.42; *p* < 0.001), objective response rate (ORR), and quality of life (QoL) [57]. OS improvement was also reported, but the confidence interval crossed the predetermined boundary for significance (*p* = 0.051); however, when adjusted for strata using the stratified Wilcoxon test, a higher degree of statistical significance was noted (*p* = 0.049). Notably, benefits were observed across all prognostic risk subgroups classified according to the Memorial Sloan Kettering Cancer Centre (MSKCC) criteria. Similarly, pazopanib demonstrated PFS (HR 0.46; *p* < 0.0001) and ORR (30% vs. 3%; *p* < 0.001) benefits over placebo in a phase III study of 435 treatment-naïve ccRCC patients [58]. The difference in OS was not statistically significant, but there was a notable crossover rate. Safety profiles of both drugs were deemed acceptable.

Optimal choice of TKI in the first-line setting remains under question, particularly relevant for those unsuitable for ICI therapy. Pazopanib was directly compared against sunitinib in a subsequent phase III study, which showed comparable PFS and OS between study arms but reported a favorable safety profile and better quality of life for pazopanib compared with sunitinib [75]. However, this study was critiqued for its flawed design and study conduct, thus proving the results uninterpretable. Another crossover study demonstrated significant patient preference for pazopanib over sunitinib [76]. Finally, cabozantinib, an oral TKI against VEGFR, MET, and AXL, demonstrated PFS benefit (HR 0.66; *p* = 0.012) over sunitinib in a phase II trial of 157 treatment-naïve, advanced-stage ccRCC patients with intermediate- to poor-risk disease according to International Metastatic Database Consortium (IMDC) criteria [77]. OS data remain immature, but the study was not powered to detect a statistically significant difference, and it is likely that phase III data will be necessary to determine true OS benefit. As such, there remains no robust data to guide whether a particular TKI is favored over another in the first-line context.

Furthermore, the role of individualized TKI dosing remains unclear particularly with sunitinib, where increased drug exposure is thought to demonstrate benefit over standard regimens. One phase II study enrolled 117 patients to receive sunitinib continuously for up to 4 weeks until prohibitive toxicity, upon which patients had a break of 7 days [78]. The trial met its primary endpoint with median PFS of 12.5 months, which when compared to the standard 4-weeks on and 2-weeks off schedule in a comparator (median PFS 8.5 months) [79] was improved, although clearly limited by cross-trial comparisons and the fact that this was a single-arm study. Intermittent TKI dosing based on treatment response has also been explored and is viable [79,80,81], although its role in standard practice is also unclear.

#### 1.3.3. Mammalian Target of Rapamycin (mTOR) Inhibitors

In addition to upregulation of VEGF and HIF secondary to inactivation of the VHL gene, activation of the mammalian target of rapamycin (mTOR) pathway also leads to increased expression of HIF-1 and was implicated as a valid target in renal cell carcinoma [82,83]. However, its use in routine clinical practice has been somewhat superseded by other targeted and ICI therapies due to better efficacy and tolerability.

In a randomized phase III study, temsirolimus alone was compared with IFN-α alone or temsirolimus plus IFN-α in patients with untreated, advanced RCC with poor risk features [84]. The study demonstrated that temsirolimus was associated with improved OS (HR 0.73; *p* = 0.008) and PFS (*p* < 0.001). OS in the combination therapy group did not differ significantly from the IFN-α group (HR 0.96; *p* = 0.70). Common toxicities associated with temsirolimus were rash, peripheral oedema, hyperglycemia, and hyperlipidemia. However, a subsequent randomized phase II study demonstrated superior outcomes with pazopanib [85]. The efficacy and sequence of oral mTOR-inhibitor everolimus in patients with advanced ccRCC was assessed in the RECORD trials. RECORD-1 was an international, double-blinded randomized phase III study which enrolled 416 advanced ccRCC patients who had progressed on VEGF therapy to received everolimus or placebo [86]. Everolimus was associated with significantly improved median PFS compared to placebo (HR 0.33; *p* < 0.01). With 80% of participants in the placebo arm crossed over to everolimus arm, there was no significant difference in median overall survival (HR 0.87; *p* = 0.162). The results established the role of everolimus in pre-treated advanced ccRCC.

The treatment sequence of everolimus and sunitinib in patients with advanced RCC (clear cell and non-clear cell) was studied in a randomized phase II study (RECORD-3) [87], but the primary endpoint of PFS non-inferiority was not met. Subsequent OS analysis also demonstrated that the HR for sequential everolimus–sunitinib/sunitinib–everolimus was above the prespecified noninferiority margin [88].

#### 1.3.4. Immune Checkpoint Inhibitors

Despite anti-angiogenic TKIs and mTOR inhibitors, most patients develop resistance to these therapies. Multiple resistant mechanisms were proposed, including dysfunction of T-cell function and signaling pathways, downregulation of antigen presentation, and barriers within the tumor microenvironment [89]. Understanding of host–tumor immune reaction led to the discovery of antibodies against immune checkpoint proteins, such as programmed death 1 (PD-1), its ligand PD-L1 and cytotoxic T-lymphocyte-associated protein 4 (CTLA-4) [90].

Ipilimumab, an immune checkpoint inhibitor against CTLA-4, was demonstrated to have clinical activity in patients with advanced ccRCC [91]. However, treatment was associated with significant toxicities, with 33% experiencing grade 3–4 immune-mediated toxicities, limiting its role as a monotherapy. On the other hand, nivolumab, an ICI against PD-1, was shown to have activity against ccRCC with a manageable safety profile in early-phase studies [92,93]. Subsequently, nivolumab was compared with everolimus in a prospective randomized phase III trial in patients with ccRCC who progressed on prior antiangiogenic therapy (CheckMate-025) [55]. Nivolumab was associated with improved OS (HR 0.73; *p* = 0.002) and response rate (OR 5.98; *p* < 0.001) compared to everolimus, with better tolerability, as the study noted rates of Grade 3 or 4 treatment-related adverse events at 19% with nivolumab opposed to 37%. This therefore established the role of ICI in pre-treated advanced ccRCC.

Combination of ipilimumab and nivolumab had demonstrated promising efficacy and higher response rates than either agent alone in multiple malignancies [94,95] including advanced ccRCC [96]. In the landmark CheckMate 214 randomized phase III study [60], nivolumab 3 mg/kg combined with ipilimumab 1 mg/kg every 3 weeks followed by maintenance nivolumab 3 mg/kg every 2 weeks was compared with sunitinib in 1096 patients with untreated advanced ccRCC. At the time of initial presentation and after 5 years of follow up (median 67.7 months), combination ICIs were associated with improved OS in the intention-to-treat (ITT) population (HR 0.72) as well as in patients with IMDC intermediate- to poor-risk disease (HR 0.68) [60,97]. ORR was also higher with combination ICIs in the ITT population (39% vs. 32%) and patients with IMDC intermediate- to poor-risk disease (*n* = 847; 42% vs. 27%). There were fewer grade 3–4 treatment-related adverse events in the combination ICI arm compared to the sunitinib arm (47.9% vs. 64.1%) [98]. Therefore, combination ipilimumab and nivolumab became a new standard of care in patients with IMDC intermediate- and poor-risk advanced ccRCC.

#### 1.3.5. Combination VEGF Monoclonal Antibody and Immune Checkpoint Inhibitor

As discussed above, TKIs and ICIs were individually active treatments in advanced ccRCC. Pre-clinical studies demonstrated that T-cell infiltration into tumors can be increased by angiogenic inhibition, thereby enhancing activity of ICIs [99]. The co-inhibition of VEGF and PD-1 enhance T-cell infiltration in a synergistic fashion, leading to early-phase studies investigating the combination approach in advanced RCC [100,101,102,103,104]. However, despite signals of efficacy, a number of studies demonstrated that combination ICI and TKI can lead to significant toxicities [101,102], suggesting the success of combination strategies depends on careful selection of anti-angiogenic agents and dosing. Herein, we summarize the outcomes of randomized phase III studies using the combination approach.

Following the discovery that bevacizumab could be efficacious in ccRCC, two randomized phase III trials studied the combination of bevacizumab with IFN-α-2a [105] and IFN-α-2b [106], demonstrating PFS benefits but no OS benefit in first-line advanced-stage ccRCC. Bevacizumab plus IFN-α-2a was associated with significantly improved PFS compared with IFN-α-2a alone (HR 0.63; *p* = 0.001), with significantly higher ORR in the bevacizumab arm (31% vs. 13%; *p* = 0.001) [105]. Similarly, bevacizumab plus IFN-α-2b demonstrated improved mPFS compared with IFN-α-2b alone (8.5 months vs. 5.2 months; HR 0.71; *p* < 0.001) [106]. These studies, although modest in efficacy, were the first combination angiogenesis and immunotherapy studies in RCC.

Bevacizumab was also assessed in combination with atezolizumab against a comparator of sunitinib in a phase III study enrolling 915 patients with clear-cell or sarcomatoid histology, of which 40% were PD-L1 positive [107]. This study met its co-primary endpoint of PFS in the PD-L1-positive population and mPFS was 11.2 vs. 7.7 months in favor of the combination (HR 0.74; *p* = 0.0217). However, OS in the ITT population was not significantly improved at the time of final analysis (minimum follow-up 40 months). Treatment-related grade 3–4 adverse events were lower in the combination arm (40 vs. 54%), with no unexpected toxicity signals.

#### 1.3.6. Combination Tyrosine Kinase Inhibitor and Immune Checkpoint Inhibitor

In addition to bevacizumab, multiple other combinations of anti-VEGF TKI and ICI regimens have found clinical success particularly in the first line ccRCC setting, with subsequent regulatory approval (see Table 1, Figure 1).

Three studies have demonstrated significant improvements in OS with combination therapy. KEYNOTE-426 was a phase III study that enrolled 861 previously untreated metastatic ccRCC patients, comparing pembrolizumab and axitinib against sunitinib [4]. 12-month OS was significantly improved in the combination arm (89.9 vs. 78.3%; HR 0.53; *p* < 0.0001). The primary endpoint, PFS, was also met (median 15.1 vs. 11.1 months; HR 0.69; *p* < 0.001). Combination therapy was also associated with an improved response rate (59.3% vs. 35.7%; *p* < 0.001). In terms of toxicity, 62.9% in the combination group and 58.1% in the sunitinib group had treatment-related AE of grade 3 or higher; the incidence of grade 3 elevation in liver enzymes in the pembrolizumab–axitinib group were higher than previously observed when each agent was used as monotherapy, which requires further examination [4]. After 42 months of follow up, superiority of PFS, OS, ORR was preserved [109].

Similarly, the CLEAR study investigated lenvatinib plus pembrolizumab, lenvatinib plus everolimus, or sunitinib alone in 1069 patients with untreated, advanced ccRCC [3]. The study met its primary endpoint, with lenvatinib–pembrolizumab showing significant improvement in PFS over sunitinib (23.9 vs. 9.2 months; HR 0.39; *p* < 0.001). Lenvatinib–everolimus also showed significantly prolonged PFS compared to sunitinib alone (HR 0.65; *p* < 0.001). OS was significantly longer in the lenvatinib–pembrolizumab arm compared to the sunitinib arm (HR 0.66; *p* = 0.005); however, there was no significant difference in OS between lenvatinib–everolimus and sunitinib (HR 1.15; *p* = 0.30). Notably, 16.1% of participants in the lenvatinib–pembrolizumab group had a complete response. In terms of safety, 82.4%, 83.1%, and 71.8% of participants experienced treatment-related adverse events of grade 3 or above in the lenvatinib–pembrolizumab arm, lenvatinib–everolimus arm, and sunitinib arm, respectively. The safety profiles of each combination therapy were consistent with that of each component as a single agent.

Nivolumab plus cabozantinib was evaluated against sunitinib in 651 patients with untreated advanced ccRCC in the CheckMate 9ER study [2]. The primary endpoint, PFS, was significantly improved with nivolumab–cabozantinib (HR 0.51; *p* < 0.001) and OS was also significantly higher (HR 0.60; *p* = 0.001). ORR assessed by independent review was also higher with nivolumab–cabozantinib (55.7 vs. 27.1%). Complete response was seen in 8%. In terms of safety, 60.6% in the nivolumab–cabozantinib group and 50.9% of the sunitinib group experienced treatment-related adverse events of grade 3 or above, and patients in the combination arm also reported better quality of life at all time points through week 91 [2].

The combination of avelumab and axitinib was compared with sunitinib in 886 patients with untreated advanced ccRCC in JAVELIN Renal 101 [108]. This phase III study also met its primary endpoint, with PFS significantly longer in the avelumab–axitinib arm than the sunitinib arm in patients with PD-L1-positive tumors (HR 0.61; *p* < 0.001) and in the overall population (HR 0.69; *p* < 0.001). ORR was significantly higher (55.2%) in the avelumab–axitinib arm than the sunitinib arm (25.5%); however, OS data were immature, and no significant benefit was seen at the time of publication. Overall, a similar proportion of patients experienced treatment-related adverse events of grade 3 or higher between the 2 groups.

The above section highlights several areas of success in therapeutic development in advanced ccRCC, but the optimal approach to treatment combinations or sequencing remains unclear. Combination ICI therapy and three different ICI and TKI combinations have demonstrated improved OS in the first-line setting. Furthermore, once patients progress on first-line combination therapies, it remains uncertain what therapies can be utilized as subsequent-line treatment after prior exposure to ICI or targeted therapies. Over time, interferon and mTOR inhibitor-based treatments have been used less frequently. Further refinement of patient selection through biomarker studies and awareness of toxicity risks will be key to differentiating specific benefits from different combinations.

### 1.4. Non-Clear-Cell Renal Cell Carcinoma

The umbrella term nccRCC encompasses many different subtypes of renal cancer that is of non-clear-cell histology. These subtypes include papillary, chromophobe, collecting-duct, medullary, and translocation RCCs [110]. Each subtype is associated with its own histopathological, genetic, and hereditary alterations, and consequently, prognosis and treatment options are very varied. For example, chromophobe RCC, which comprises approximately 5% of RCC and usually presents with early-stage disease, tends not to respond to ICI therapy [111,112,113].

The pathogenesis of ccRCC, such as upregulation of VEGF and HIF via the VHL or the mTOR pathways, is not applicable in nccRCC. Overexpression of pathways such as mesenchymal–epithelial transition factor (MET) signaling have been explored in subtypes such as papillary RCC, which accounts for 10–15% of RCC [113]. Due to the diverse histology and rarity of each individual subtype, nccRCCs are traditionally underrepresented in clinical trials and their treatments are not well-studied [114].

In the everolimus versus sunitinib prospective evaluation in metastatic non-clear-cell renal cell carcinoma (ESPN) randomized phase II trial [115], patients with advanced nccRCC, or ccRCC with ≥20% sarcomatoid features were randomized to receive sunitinib or everolimus with crossover at disease progression. Based on recommendation from the data-monitoring committee, the study was closed to recruitment early after accrual of 73 patients. The mPFS was numerically better with the sunitinib arm (6.1 months) than everolimus (4.1 months) but the difference was not statistically significant (*p* = 0.6). At final analysis, the mOS was 16.2 and 14.9 months with sunitinib and everolimus, respectively (*p* = 0.18). In the exploratory analyses, among the 49 patients whose tumor had no sarcomatoid features, median OS for patients receiving first-line sunitinib and first-line everolimus were 31.6 months and 10.5 months, respectively (*p* = 0.075).

In the randomized phase II everolimus versus sunitinib for patients with metastatic non-clear-cell renal carcinoma study (ASPEN), patients with untreated advanced papillary, chromophobe, or unclassified nccRCC were randomized to sunitinib or everolimus. Sunitinib was associated with significantly prolonged mPFS compared with everolimus (8.3 months vs. 5.6 months; HR 1.41; *p* = 0.16). However, there was significant heterogeneity by histology and prognostic risk groups. There was no significant difference in overall survival between the two groups (HR 1.12; *p* = 0.6). Taken together, the ESPN and ASPEN trials demonstrated that sunitinib and everolimus provide modest benefits in patients with advanced nccRCC, highlighting the need for better treatments in this population.

ICI therapy has been explored as monotherapy and in combination with other agents. A total of 83 patients with nccRCC were enrolled in a phase II trial to receive nivolumab every 2 weeks (part 1), following which they were switched to nivolumab in combination with ipilimumab upon disease progression (part 2) [116]. The primary endpoint from part 1, ORR, was 17% with a median duration of response of 21 months and a similar adverse effect profile to other ICI therapy studies. Another phase II study, SUNNIFORECAST (NCT03075423), is also currently exploring the role of the combination of nivolumab and ipilimumab in advanced nccRCC.

More recently, the results of a prospective phase II study evaluating cabozantinib and nivolumab in nccRCC was presented in abstract form [117]. In this study, patients were divided into two cohorts. Cohort 1 included papillary, unclassified, or translocation associated RCC; cohort 2 included chromophobe RCC. A total of 40 patients were recruited to cohort 1 and 7 patients were recruited to cohort 2. In cohort 1, 65% were treatment naïve. ORR in this cohort was 48%, mPFS was 12.5 months and mOS was 28 months. None of the patients in cohort 2 had a response. These results suggest that the combination of cabozantinib and nivolumab has activity in patients with papillary, translocation and unclassified nccRCC.

The role of the combination of bevacizumab and atezolizumab in advanced RCC with variant histology was evaluated in a single-arm phase II study [111]. A total of 60 patients with nccRCC with or without sarcomatoid differentiation, or ccRCC with ≥20% sarcomatoid differentiation, were included for analysis, of which 70% had nccRCC. A total of 65% were treatment naïve. ORR was 33% in the ITT population. ORR for ccRCC with ≥20% sarcomatoid differentiation and nccRCC were 50% and 26%, respectively. The mPFS was 8.3 months in the overall study population; PFS according to the different histologic subgroups was not reported. Interestingly, it appeared as though PD-L1 status was predictive of treatment response in nccRCC, as the ORR in PD-L1-positive and PD-L1-negative patients was 67% and 14%, respectively (*p* = 0.02).

MET inhibition was evaluated in the phase II PAPMET study, which enrolled 152 patients with metastatic papillary RCC and randomized them to receive savolitinib, crizotinib, sunitinib, or cabozantinib [118]. After ceasing savolitinib and crizotinib arms due to futility, cabozantinib was demonstrated to show PFS benefit over sunitinib (median 9 months vs. 5.6 months; one-sided *p* = 0.019). Grade 3–4 adverse events occurred in 74% and 69% of patients receiving cabozantinib and sunitinib, respectively.

In summary, although therapeutic benefit has been demonstrated amongst some subtypes of nccRCC with various drug classes, this group of patients remains difficult to obtain robust evidence on due to disease rarity. Several clinical trials are underway in this space (Table 2).

### 1.5. Opportunities for Advancement in Renal Cell Carcinoma Drug Development

Although there has been a rapid succession of positive combination studies in advanced-stage RCC utilizing immunotherapy and angiogenesis inhibitors, therapeutic resistance and consequent disease-specific mortality remains an unresolved issue requiring urgent attention.

Several questions remain unanswered regarding these strategies particularly pertaining to patient selection, clinical trial design, and novel therapeutic targets. These will become increasingly relevant over time, and it is likely that biomarker and drug discovery advances will lead to more complex patient subclassification and treatment regimens.

Patient selection remains important. In KEYNOTE-426, pembrolizumab-axitinib demonstrated an ORR of 59.3%, yet there were still 47 patients (10.9%) who demonstrated progressive disease as best response [119]. In this study, pre-specified and post-hoc preliminary biomarker subgroup analyses did not demonstrate any unexpected findings. Higher ORR and survival rates were seen in PD-L1-positive patients and those in the intermediate-poor IMDC risk subgroups, similar to other trials involving ICI therapies [2,3,60,108]. Ongoing trials in ccRCC are listed in Table 3. Translational endpoint data from these studies are highly anticipated as they may provide insight into which biomarkers are predictive of treatment response or resistance.

The phase II study BIONIKK (NCT02960906) is currently ongoing and explores the feasibility and clinical efficacy of personalized therapy guided by RNA sequencing in treatment-naïve, advanced-stage ccRCC [120]. A total of 202 patients were randomized into four groups to receive nivolumab, nivolumab–ipilimumab, or a TKI (pazopanib or sunitinib) based on angiogenic and immune signatures. Initial outcomes after a median follow up of 16 months between treatment arms were noted based upon biomarker profile groups [121]. Whilst these analyses are still relatively immature, at the time of writing this was the only presented prospective RCC study that demonstrated variable clinical outcomes based upon gene expression signatures.

Increasingly, adaptive clinical trial designs are being adopted to allow multi-cohort expansions for those in rarer subgroups. As highlighted above, nccRCC is a heterogeneous entity in which each histological subtype demonstrates unique disease biology and varied responses to systemic therapies. Due to the rarity of each subtype, accruing adequately powered randomized trials remains a challenge, although some success has been noted, such as the PAPMET study [118]. Appropriately, there are a number of trials in progress involving TKI and ICI combinations (Table 3), which include histology-specific nccRCC cohorts. These results will be paramount in defining the evolving standard of care therapy and obtaining regulatory approval in nccRCC subtypes.

One biomarker of interest across many tumor types is the effects of gut microbiota on anti-cancer therapy, with a particular focus on ICI therapies and antibiotic use. A prospective phase II study in France collected fecal samples from 69 patients as part of a translational substudy and compared samples from healthy volunteers as controls [122]. Metagenomic data from whole genome sequencing were assessed to look for prior antibiotic or TKI exposure and correlated with clinical response data to nivolumab. Interestingly, recent antibiotic use (*n* = 11; 16%) decreased ORR from 28% to 9% (*p* < 0.03) [122]. Prior TKI use significantly altered fecal microbiota composition with specific bacterial overgrowth demonstrated with axitinib, sunitinib, and cabozantinib in particular; some bacteria were thought to be immunostimulatory and, hypothetically, could improve clinical outcomes with ICI therapies. At this stage, evidence of gut dysbiosis due to prior therapies in RCC are hypothesis-generating, and further work is required to explore the interactions with the tumor microenvironment to optimize clinical outcomes in RCC patients.

Novel anti-angiogenesis therapeutic targets are currently being explored through monotherapy and combination studies, with varied success. Targeting of the angiopoietin pathway with trebananib with or without bevacizumab was unfortunately not clinically significant, and further testing of this compound has halted [123]. However, with the regulatory approval of belzutifan, the first HIF inhibitor, in advanced ccRCC with VHL disease, its clinical activity is being evaluated further in early-phase, multi-cohort, umbrella studies involving various combinations of ICI and targeted therapies (Table 2). A phase II dose expansion cohort of belzutifan and cabozantinib in 52 previously treated, advanced ccRCC patients demonstrated an ORR of 22% and a median PFS of 16.8 months (9.2 to not reached) [124], with safety profiles not dissimilar to that of each individual agent. It remains to be seen whether longer follow up and different combinations will demonstrate meaningful clinical benefits for these patients.

## 2. Conclusions

Within the last few decades, scientific advancements within oncology and drug development have dramatically altered the therapeutic landscape of advanced-stage RCC. Based on the hallmarks of cancer development, interactions within the tumor microenvironment have fueled interest into various successful trials examining the synergy behind anti-angiogenic therapies and immunotherapies, and oncology clinicians are now faced with a plethora of combinations to choose from. Despite these victories, cancer-specific mortality remains high, and further work needs to be conducted to differentiate patients further to predict who is likely to demonstrate treatment response or resistance. Other challenges that require further exploration include drug-class resistance as well as rare tumor histologies including specific nccRCC groups. Through utilizing flexible clinical trial designs, integral biomarker analyses, and adoption of novel combination therapeutics, it is hoped that we can improve survival outcomes, therapeutic safety, and quality of life for patients with advanced RCC.

## Figures and Tables

**Table 1:** Presented phase III studies regarding combination immune checkpoint inhibitor and anti-angiogenic therapy trials in previously untreated, advanced-stage clear-cell renal cell carcinoma.

Trial	Study Population	Drugs	Enrollment	Primary Outcome Measures	Key Outcomes
IMmotion151 [107] NCT02420821	Previously untreated, advanced RCC with clear-cell or sarcomatoid histology Any IMDC risk group	**Arm 1:** Atezolizumab 1200 mg plus bevacizumab 15 mg/kg IV Q3W **Arm 2**: Sunitinib 50 mg PO daily, 4 weeks on treatment followed by 2 weeks off treatment	915	PFS in the PD-L1-positive population and OS in ITT population	mPFS in PDL1 + ve patients: atezolizumab + bevacizumab vs. sunitinib: 11.2 mo vs. 7.7 mo. HR 0.74; 95%CI 0.57–0.96; *p* = 0.0217 OS in ITT (interim): atezolizumab + bevacizumab vs. sunitinib: 43% vs. 42%. HR 0.93; 95%CI 0.76–1.14; *p* = 0.4751
KEYNOTE-426 [4] NCT02853331	Previously untreated, advanced RCC with clear-cell component with or without sarcomatoid features Any IMDC risk group	**Arm 1:** Pembrolizumab 200 mg IV Q3W and axitinib 5 mg PO twice daily **Arm 2:** Sunitinib 50 mg PO daily, 4 weeks on treatment followed by 2 weeks off treatment	861	PFS	mPFS: pembrolizumab + axitinib vs. sunitinib: 15.1 mo vs. 11.1 mo. HR 0.69; 95%CI 0.57–0.84; *p* < 0.001
JAVELIN Renal 101 [108] NCT02684006	Previously untreated, advanced RCC with a clear-cell component	**Arm 1:** Avelumab 10 mg/kg IV Q2W plus axitinib 5 mg PO BD **Arm 2:** Sunitinib 50 mg PO daily, 4 weeks on treatment followed by 2 weeks off treatment	886	PFS and OS in patients with PD-L1-positive tumors	63.2% had PD-L1-positive tumors. mPFS in PD-L1-positive tumors: avelumab + axitinib vs. sunitinib: 13.8 mo vs. 7.2 mo. HR 0.61; 95%CI 0.47–0.79; *p* < 0.001. OS in PD-L1-positive tumors: avelumab + axitinib vs. sunitinib: HR 0.82; 95%CI 0.53–1.28; *p* = 0.38
CLEAR [3] NCT02811861	Previously untreated, advanced RCC with a clear-cell component Any IMDC risk group	**Arm 1:** Lenvatinib 18 mg PO daily plus everolimus 5 mg PO daily **Arm 2:** Lenvatinib 20 mg PO daily plus pembrolizumab 200 mg IV Q3W **Arm 3:** Sunitinib 50 mg PO daily, 4 weeks on treatment followed by 2 weeks off treatment	1069	PFS	Lenvatinib + pembrolizumab vs. sunitinib: 23.9 mo vs. 9.2 mo; HR 0.39; 95%CI 0.32–0.49; *p* < 0.001) Lenvatinib plus everolimus vs. sunitinib: 14.7 mo vs. 9.2 mo; HR 0.65; 95%CI 0.53–0.80; *p* < 0.001)
CheckMate 9ER [2] NCT03141177	Previously untreated, advanced RCC with a clear-cell component Any IMDC risk group	**Arm 1:** Nivolumab 240 mg IV Q2W plus cabozantinib 40 mg PO daily **Arm 2:** Sunitinib 50 mg PO daily, 4 weeks on treatment followed by 2 weeks off treatment	651	PFS	Nivolumab + cabozantinib vs. sunitinib: 16.6 mo vs. 8.3 mo; HR 0.51; 95%CI 0.41–0.64; *p* < 0.001)

Abbreviations: RCC = renal cell carcinoma; IMDC = international metastatic RCC database consortium; Q3W = every 3 weeks; PFS = progression-free survival; mPFS = median progression-free survival; PD-L1 = programmed cell death ligand-1; ITT = intention to treat; OS = overall survival; PO = per oral; IV = intravenous; 95%CI = 95% confidence interval; Q2W = every 2 weeks; HR = hazard ratio.

**Table 2:** Current combination immune checkpoint inhibitor and anti-angiogenic therapy trials in non-clear-cell renal cell carcinoma, as obtained from clinicaltrials.gov.

Trial	Phase	Study Population	Drugs	Estimated Enrollment	Primary Outcome Measures	Estimated Primary Study Completion
NCT02724878	II	Unresectable/metastatic nccRCC Untreated or previously treated	Atezolizumab 1200 mg IV Q3W + bevacizumab 15 mg/kg IV Q3W	60	ORR	April 2021 *
LENKYN NCT04267120	II	Unresectable/metastatic nccRCC Previously untreated	Pembrolizumab 200 mg IV Q3W + lenvatinib 20 mg daily PO	34	ORR	July 2024
KEYNOTE-B61 NCT04704219	II	Unresectable/metastatic nccRCC Previously untreated	Pembrolizumab 400 mg IV Q6W + lenvatinib 20 mg daily PO	152	ORR	August 2024
CA209-9KU NCT03635892	II	Unresectable/metastatic nccRCC 0–1 previous lines of treatment	Cabozantinib 40 mg daily PO + nivolumab 240 mg Q2W	97	ORR	August 2022
NCT04413123	II	Unresectable/metastatic nccRCC Untreated or previously treated	Cabozantinib PO daily + nivolumab IV Q3W + ipilimumab IV Q3W 4 cycles, then maintenance cabozantinib and nivolumab	40	ORR	December 2021
NCT04385654	II	Metastatic nccRCC T2–4N0 or TxN1+ or nuclear grade > 3	Toripalimab 240 mg Q3W IV + axitinib 5 mg BD PO for 6 weeks followed by resection of primary tumor	40	MPR, pCR, pNR	December 2021
ICONIC NCT03866382	II	Rare GU tumors (nccRCC cohorts include sarcomatoid, RMC, CDC, papillary, chromophobe, tRCC)	Nivolumab Q3W IV + ipilimumab Q3W IV + cabozantinib daily PO for 4 cycles, followed by maintenance nivolumab + cabozantinib	224	ORR	February 2023
CONTACT-03 NCT04338269	III	Unresectable/metastatic RCC, incl. nccRCC cohort (papillary, chromophobe, unclassified, sarcomatoid)	Arm 1: Atezolizumab 1200 mg IV Q3W + cabozantinib 60 mg daily PO Arm 2: Cabozantinib 60 mg daily PO	500	PFS, OS	December 2022
NCT03595124	II	Unresectable/metastatic tRCC	Arm 1: Axitinib BD PO + nivolumab Q2W IV Arm 2: Axitinib BD PO Arm 3: Nivolumab Q2W IV	70	PFS	Suspended (poor accrual)

Abbreviations: nccRCC = non-clear-cell renal cell carcinoma; IV = intravenous; Q3W = every 3 weeks; ORR = overall response rate as per RECIST v1.1; PO = per oral; Q6W = every 6 weeks; Q2W = every 2 weeks; BD = twice daily; MPR = major pathologic response; pCR = pathological complete response; pNR = pathological no response; GU = genitourinary; RMC = renal medullary carcinoma; CDC = collecting-duct carcinoma; tRCC = translocation-positive renal cell carcinoma; PFS = progression-free survival; OS = overall survival. * Preliminary results for NCT02724878 have been published in 2020 in the Journal of Clinical Oncology, with ORR 50% in patients with clear-cell RCC with sarcomatoid differentiation and 26% in those with other variants of non-clear-cell histology [111].

**Table 3:** Trial in progress for combination immune checkpoint inhibitor and anti-angiogenic therapies in advanced-stage clear-cell renal cell carcinoma, as obtained from clinicaltrials.gov.

Trial	Phase	Population	Treatment	Estimated Enrollment	Primary Endpoints	Estimated Primary Study Completion
COSMIC 313 NCT03937219	III	Previously untreated, unresectable/metastatic renal cell carcinoma with a clear-cell component IMDC intermediate or poor risk	Arm 1: Cabozantinib + nivolumab + ipilimumab (4 doses) followed by cabozantinib + nivolumab Arm 2: Cabozantinib-matched placebo + nivolumab + ipilimumab (4 doses) followed by cabozantinib-matched placebo + nivolumab	840	PFS	November 2021
CONTACT-03 NCT04338269	III	Advanced, untreated RCC ccRCC and nccRCC cohorts	Arm 1: Atezolizumab 1200 mg IV Q3W + cabozantinib 60 mg PO daily Arm 2: Cabozantinib 60 mg PO daily	500	PFS, OS	December 2022
PEDIGREE NCT03793166	III	Unresectable/metastatic RCC with clear-cell component, including patients with sarcomatoid features IMDC intermediate and poor risk	Induction ipilimumab + nivolumab. In patients with non-CR/non-PD: Arm 1: maintenance nivolumab alone Arm 2: maintenance nivolumab plus cabozantinib	1046	OS	September 2022
TiNivo-2 NCT04987203	III	Advanced RCC with a clear component, progressed during or following at least 6 weeks of ICI treatment in the first- or second- line setting	Arm 1: Nivolumab IV Q4W plus tivozanib 1.34 mg PO daily, 3 weeks on treatment followed by 1 week off treatment Arm 2: Tivozanib 1.34 mg PO daily, 3 weeks on treatment followed by 1 week off treatment	326	PFS	July 2024
TIDE-A NCT04698213	II	Metastatic RCC with predominately clear-cell subtype with primary tumor resected	Axitinib 5 mg PO BD + avelumab 10 mg/kg IV Q2W	75	ORR	September 2023
NCT03172754	I/II	Untreated, advanced RCC with predominately clear-cell subtype	Axitinib + nivolumab	98	TRAE, ORR	April 2023
NCT03149822	I/II	Advanced or metastatic RCC	Cabozantinib + pembrolizumab	45	ORR	December 2020
MK-3475-03A NCT04626479	I/II	Untreated, locally advanced or metastatic clear-cell RCC	Pembrolizumab + favezelimab Pembrolizumab + belzutifan Pembrolizumab + MK-4830 Pembrolizumab + lenvatinib Lenvatinib + belzutifan Pembrolizumab + quavonlimab	390	TRAE	June 2025
MK-3475-03B NCT04626518	I/II	Locally advanced or metastatic ccRCC	Pembrolizumab + favezelimab Pembrolizumab + belzutifan Pembrolizumab + MK-4830 Pembrolizumab + lenvatinib Lenvatinib + belzutifan Pembrolizumab + quavonlimab	370	TRAE	May 2025
NCT03634540	II	Locally advanced or metastatic ccRCC Cohort 1: previously treated Cohort 2: immunotherapy-naïve	Belzutifan 120 mg PO daily + cabozantinib 60 mg PO daily	118	ORR	August 2025

Abbreviations: RCC = renal cell carcinoma; IMDC = international metastatic RCC database consortium; PFS = progression-free survival; IV = intravenous; PO = per oral; OS = overall survival; non-CR = non-complete response; non-PD = non-progressive disease; ICI = immune checkpoint inhibitor; ccRCC = clear-cell RCC; nccRCC = non-clear-cell RCC; Q2W = every 2 weeks; Q3W = every 3 weeks; Q4W = every 4 weeks; ORR = overall response rate; TRAE = treatment-related adverse events.
